# In this month’s Bulletin

**DOI:** 10.2471/BLT.24.000424

**Published:** 2024-04-01

In this month’s editorial section, Manjulaa Narasimhan et al. (226) describe three decades of progress and setbacks since the first international conference on population and development. Stuart Gietel-Basten (227) explores contemporary demographic challenges and population policies.

Gary Humphreys (230–231) reports on tobacco advertising regulations in the context of social media. De Wet Swanepoel talks to Gary Humphreys (232–233) about digital health technologies being used to improve access to hearing health care.

## China

### Fertility intentions and fecundity 

Qin Li et al. (244–254) analyse findings from a nationally-representative survey. 

### Snakebite prevention

Lanfen He et al. (234–243) assess snakebite knowledge and practices across ten provinces.

## Côte d’Ivoire, Kenya, United Republic of Tanzania 

### Schistosomiasis prevalence 

Ryan E Wiegand et al. (265–275) examine trial data to extract age-group associations.

## Malawi

### Open tibia fractures

Alexander Thomas Schade et al. (255–264) evaluate measures to improve case management. 

## Global

### Vaccine coverage and childhood survival 

Haijun Zhang et al. (276–287) quantify two decades of progress.

### Leprosy diagnostics

Petra Kukkaro et al. (288–295) introduce a new target product profile.

